# Syrphidae of Southern Illinois: Diversity, floral associations, and preliminary assessment of their efficacy as pollinators

**DOI:** 10.3897/BDJ.8.e57331

**Published:** 2020-10-29

**Authors:** Jacob L Chisausky, Nathan M Soley, Leila Kassim, Casey J Bryan, Gil Felipe Gonçalves Miranda, Karla L Gage, Sedonia D Sipes

**Affiliations:** 1 Southern Illinois University Carbondale, School of Biological Sciences, Carbondale, IL, United States of America Southern Illinois University Carbondale, School of Biological Sciences Carbondale, IL United States of America; 2 Iowa State University, Department of Ecology, Evolution, and Organismal Biology, Ames, IA, United States of America Iowa State University, Department of Ecology, Evolution, and Organismal Biology Ames, IA United States of America; 3 Canadian National Collection of Insects, Arachnids and Nematodes, Ottawa, Canada Canadian National Collection of Insects, Arachnids and Nematodes Ottawa Canada; 4 Southern Illinois University Carbondale, College of Agricultural Sciences, Carbondale, IL, United States of America Southern Illinois University Carbondale, College of Agricultural Sciences Carbondale, IL United States of America

**Keywords:** Syrphidae, hover flies, flower flies, syrphid richness, Southern Illinois, pollinators, pollen load, species accumulation curve, Toxomerus

## Abstract

Syrphid flies (Diptera: Syrphidae) are a cosmopolitan group of flower-visiting insects, though their diversity and importance as pollinators is understudied and often unappreciated. Data on 1,477 Syrphid occurrences and floral associations from three years of pollinator collection (2017-2019) in the Southern Illinois region of Illinois, United States, are here compiled and analyzed. We collected 69 species in 36 genera off of the flowers of 157 plant species. While a richness of 69 species is greater than most other families of flower-visiting insects in our region, a species accumulation curve and regional species pool estimators suggest that at least 33 species are yet uncollected. In order to further the understanding of Syrphidae as pollinators in the Southern Illinois region, we produced a NMDS ordination of floral associations for the most common syrphid species. The NMDS did not sort syrphid species into discrete ecological guilds, and syrphid floral associations generally fit those predicted by traditional pollination syndromes. We also conducted a preliminary analysis of the pollen-carrying capacity of different syrphid taxa, which found several *Eristalis* species to carry pollen loads comparable to the European Honey Bee, *Apismellifera*, and showed significant differences in the pollen-carrying capacity of various syrphid species. Notably, the extremely common genus *Toxomerus* and other small Syrphinae species carried very little pollen, while large and pilose Eristalinae species carried large pollen loads.

## Introduction

Syrphidae, known as syrphids, hover flies, or flower flies, is one of the largest families of flies (Diptera), represented by almost 6,000 described species worldwide and 812 in the United States and Canada (Miranda et al. 2013). Syrphids are common flower visitors, though their role in pollinator communities is understudied and often unappreciated (Inouye et al. 2015, Klecka et al. 2018, Rader et al. 2015). Syrphids are pollinators of many wild plants (Ssymank et al. 2008), in some cases as important as bees (Forup et al. 2007, Levesque and Burger 1982, Ornduff 1975, Scribailo and Posluszny 1984). Compared to bees, syrphids as a group tend to exhibit higher degrees of generalization (Klecka et al. 2018, Lucas et al. 2018), a propensity to visit flowers considered anemophilous (Ssymank and Gilbert 1993, Inouye et al. 2015, Holloway 1976), and a capacity for temporary floral constancy similar to that of bees (Inouye et al. 2015, Raguso 2020). Syrphid adults feed almost exclusively on pollen, nectar, or honeydew (Rotheray and Gilbert 2011), and at least one species is known to possess branched palynophilic hairs and pollen combing behavior similar to that of bees (Holloway 1976). Because syrphids do not provision their offspring in a nest as do bees, syrphids are able to range over more of the landscape and may carry pollen longer distances than bees (Lysenkov 2009, Rader et al. 2011). However, the wide diversity in ecology and morphology makes generalizations about the efficacy of Syrphidae as pollinators difficult (Raguso 2020).

Evaluating the efficacy of flower-visiting taxa as pollinators can be difficult and labor-intensive. Often, visitation data alone is used as an indicator of pollinator efficacy, but this assumption may lead to overestimation of the importance of abundant species that do not actually transport, or transport little, pollen between conspecific flowers (King et al. 2013). As such, some measure of pollinator 'quality' is necessary in order to evaluate the importance of different pollinators to plant reproduction (Herrera 1987). Factors influencing pollinator quality include visitation rate, floral constancy, and the amount of pollen carried on a visitor's body (Herrera 1987, Herrera 1989, Tepedino et al. 2011, Erhardt 2008). A species' mean pollen load size has been found to correlate positively with pollen deposition on stigma (Howlett et al. 2011). In general, flies carry less pollen than bees (Orford et al. 2015, Inouye et al. 2015). While some authors have suggested that the abundance of flies as flower visitors may make up for their inefficiency as pollinators, this may not be true, as visitors that consume floral resources with little or no pollination services may negatively impact plant pollination. Because pollinator efficacy is variable within Syrphidae (Raguso 2020), measures of pollinator quality should be conducted on a per-species basis.

Comprising six level IV US EPA Ecoregions (Woods et al. 2006), Southern Illinois is an area of high biodiversity within the Midwestern US (Stein et al. 2000). Syrphids, however, have historically been little sampled in the Southern Illinois region, with just 8 museum records compiled on GBIF for the 16 southernmost counties of Illinois as of May 2020 (GBIF.org 2020a). The objectives of this paper are 1) to report on Syrphidae diversity in Southern Illinois using data of floral visitors from a region-wide pollinator inventory, and 2) to develop a baseline of understanding of the efficacy of Syrphidae as pollinators of wild plants in Southern Illinois by establishing measures of both the abundance and potential pollinator quality of syrphid species in the region. To our knowledge, this is the first study to provide a regional inventory of syrphid species in Southern Illinois.

## Material and methods

### Collection methods

Collection methodology was consistent for each of the three studies contributing data, and followed standardized procedures for bee sampling (Droege et al. 2016), with slight modifications made to accommodate the objectives of the studies. Collection events all consisted of targeted hand-netting of floral visitors plus pan trapping. These events were supplemented with additional opportunistic hand-netting. The use of both pan trap and hand-net methods has been shown to be complementary and offset the taxonomic biases of each method alone (Baum and Wallen 2011). Hand-netting was conducted for 80 person-minutes per collection event. Flower-visiting hymenopterans, dipterans, coleopterans, and lepidopterans were collected with aerial insect nets and euthanized in cyanide kill jars. Insects were kept separate by floral associations. Netting was conducted primarily during clear, sunny days. All netting was carried out between 7:00 and 17:00. Plants from which floral visitors were collected were identified to species using published keys of Mohlenbrock (2014).

Pan traps were 7 cm diameter polypropylene bowls (DART manufacturer, stock number 325PC) painted fluorescent blue, yellow, or white and filled with a dilute detergent solution (Dawn Blue dish soap). Traps were set out in sets of three along a transect at a spacing of 10 m. Each set consisted of a blue, a yellow, and a white bowl placed along a line perpendicular to the transect and spaced 5 m apart. Pan trap sets were set along two 50 m transects. Pan traps were left from 4-6 hours during daylight hours, all between 7:15 and 18:00.

### Study sites & dates

**Figure 1.**
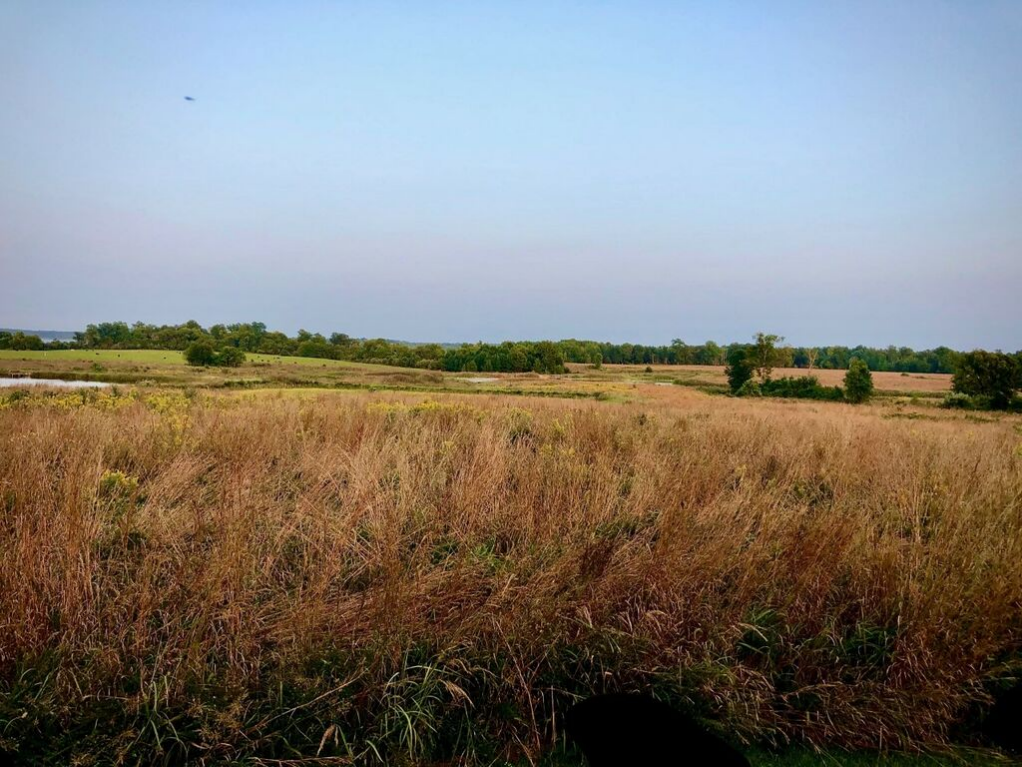
Bass Ponds, a wet habitat typical of Crab Orchard National Wildlife Refuge. Photo credits Daniel Presley.

**Figure 2.**
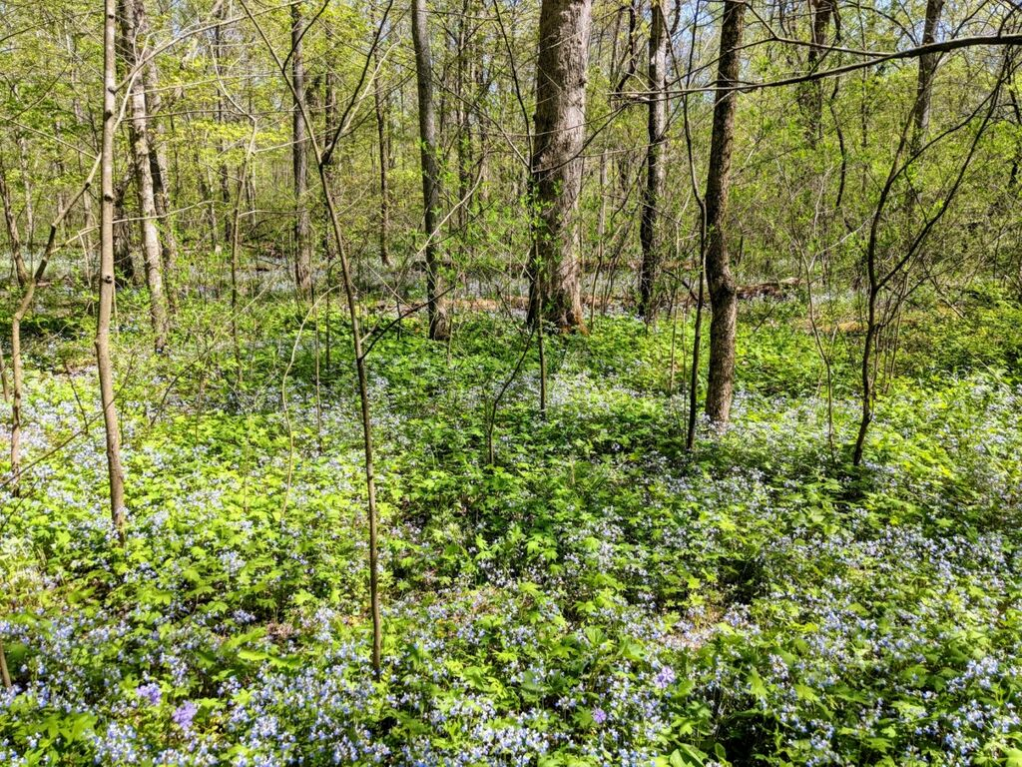
Rocky Bluff (Crab Orchard National Wildlife Refuge) in early spring, a typical forested habitat of Southern Illinois. *Collinsiaverna* (Scrophulariaceae) in bloom. Photo credits LK.

**Figure 3.**
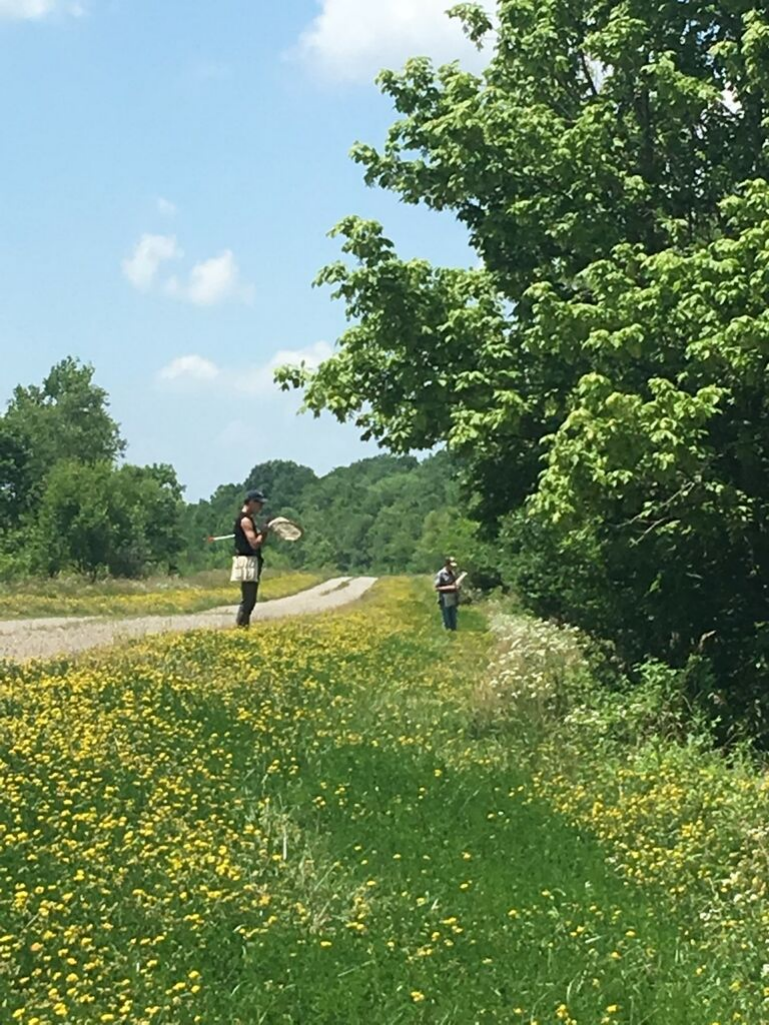
Typical grassland-forest habitat matrix of Sparta Training Area. *Lotuscorniculatus* (Fabaceae) in bloom. JLC and Daniel Crosby pictured hand-netting floral visitors. Photo credits Carmen Burkett.

**Figure 4.**
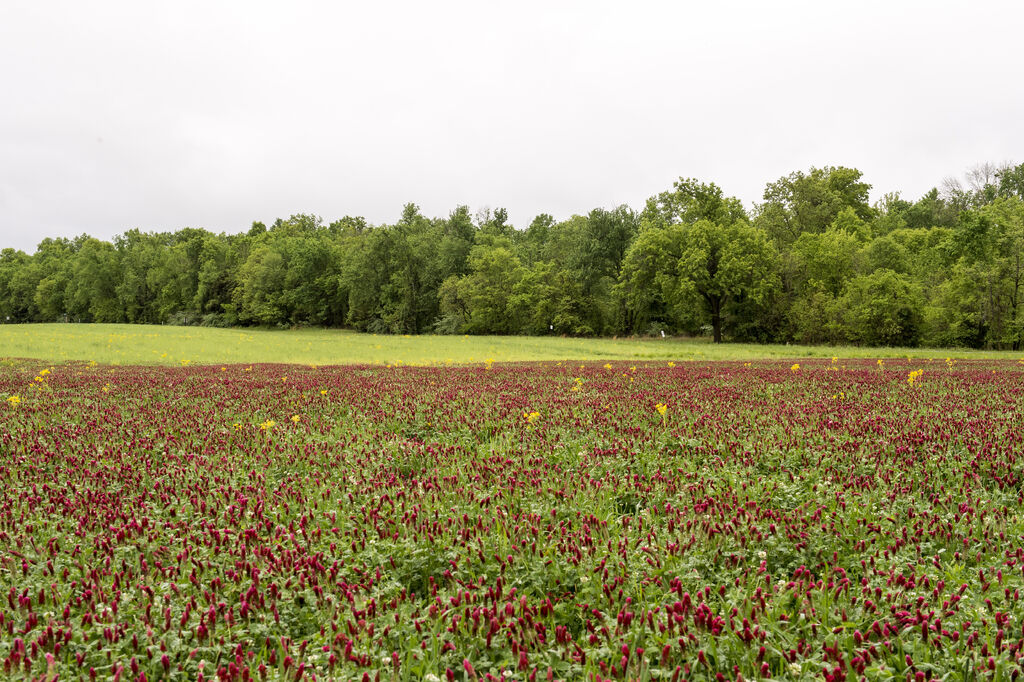
A field sampled for floral visitors during the cover crop study. The field is planted with *Trifoliumincarnatum* (flowering at time of photo), *T.repens*, and *T.pratense* (Fabaceae). Photo credits SIUC photographer Russell Bailey.

All specimens reported here were collected during surveys of all flower-visiting taxa. We sampled throughout the southernmost eleven counties of Illinois as well as Randolph county. Sampling focused on federally managed lands (Shawnee National Forest and Crab Orchard Wildlife Refuge) but also included state and private lands. Sites were stratified with respect to land use and major habitat types, and included upland and bottomland hardwood forests, open areas, roadsides, agricultural fields, reclaimed strip mines, limestone glades, and wetlands. The majority of the specimens were collected during three studies: a 2017-2019 regional inventory of flower-visiting insects of Southern Illinois focused on the USFS Shawnee National Forest and USFWS Crab Orchard Wildlife Refuge (Figs 1, 2), a 2017 study of pollinators on agricultural weeds and clover cover crops in agrosystems within Crab Orchard Wildlife Refuge in Williamson County (Fig. 4), and a 2019 inventory of the Illinois National Guard Sparta Training Area in Randolph County, IL (Fig. 3). Taxa other than Syrphidae collected during these studies will be reported elsewhere.

**Figure 5.**
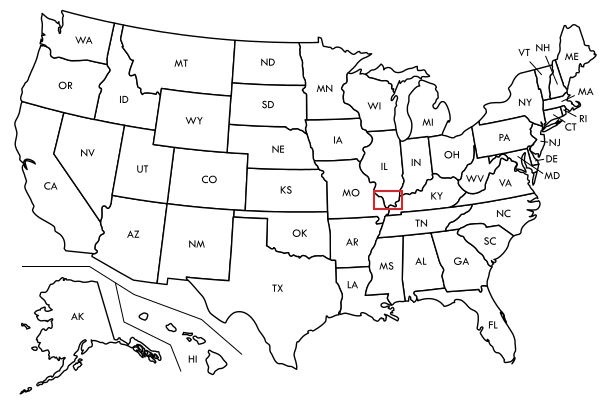
Map of US showing the study region outlined in red.

**Figure 6.**
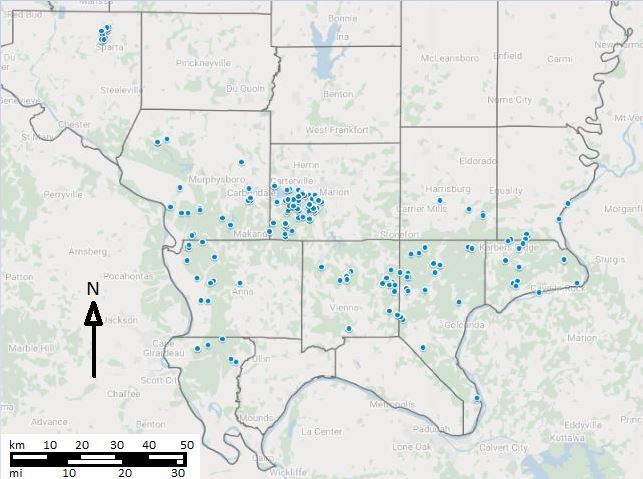
Location of sites in Southern Illinois, United States, from which syrphids were collected. Many sites were revisited several times. Map created in Google My Maps.

Floral visitors were collected from April-September of 2017, February-September of 2018, and March-July of 2019. Over the three years of collection, 292 sites were visited and 756 collection events conducted, with 55% of these events conducted for the regional inventory, 40% for the agricultural study, and 5% for the Sparta Training Area inventory. Syrphids were collected at 222 of the sites and 445 collection events (Figs 5, 6; Suppl. material 1). The 70 sites and 311 collection events that did not yield any syrphids are not included in these analyses.

### Species identification

Specimens were identified to species by JLC and GFGM using published keys of Skevington et al. (2019) and Miranda et al. (2013). Two percent of specimens were identified only to genus level (female *Sphaerophoria* and *Paragus*), and 6% were unidentified due to damage. Species-level circumscriptions follow Skevington et al. (2019). All collected specimens are deposited at the Southern Illinois University Carbondale.

### Species accumulation and species pool estimators

To determine if syrphids were sufficiently surveyed to capture the species richness of Southern Illinois, a species accumulation curve was generated based on individuals sampled using the rarefaction method (rationale in Gotelli and Colwell (2001). Specimens unidentified to species were not included in this analysis. The regional species pool was estimated by first-order jackknife and bootstrap estimators. Jackknife estimators have been shown to perform better than other estimators where a small proportion of the total species richness has been sampled (Fattorini 2013), as is suggested by the species accumulation curve for this study. These analyses were conducted in the package "vegan" version 2.5-4 in R version 3.5.1 (Oksanen et al. 2019).

### Pollen load estimation

To survey the potential efficacy of syrphids as pollinators, we assessed pollen carried on specimens using a modification of the methods of Tepedino et al. (2011). Syrphid pollen coverage was estimated for eight regions of the body: dorsal head, anterior head, ventral head, dorsal thorax, legs, ventral thorax, dorsal abdomen, and ventral abdomen. Syrphids were examined under a dissecting microscope and the pollen coverage for each region was scored either 0 (no pollen grains present on region), 1 (1-several pollen grains on region), 2 (pollen grains separated by >1mm), 3 (pollen grains separated by <1mm), 4 (near complete pollen coverage of region) or 5 (multiple layers of pollen covering region). For the two most abundant species, *Toxomerusmarginatus* and *T.geminatus*, a subsample of 39 and 25 undamaged specimens were selected for examination, respectively. For all other species, all specimens collected off flowers were examined (386 specimens total). A selection of 30 specimens of *Apismellifera* (European Honey Bee) were also examined for comparison to the syrphids. Scopal pollen was ignored in *A.mellifera* pollen scoring.

To test for significant differences in pollen load size between species, a weighted mean score for each specimen was calculated by downweighting the scores for the three head regions by 1/3, and then averaging all 8 scores. Downweighting the head regions corrected the bias of having three head regions versus two abdominal and thoracic regions. These weighted mean scores were used to run a Kruskal-Wallis non-parametric test and post-hoc Bonferroni corrected pairwise Wilcoxon Rank Sum Tests in R (alpha=.05). Only those species with >6 specimens examined were included in the analysis (18 species and 338 specimens). *Apismellifera* was not included in statistical analyses.

### Floral association ordination

A non-metric multidimensional scaling (NMDS) ordination of syrphid species by floral association genera was produced to identify guilds of floral visitors (Bray-Curtis distance). The 18 most abundant floral visiting syrphid species were included, excepting *Toxomerusjussiaeae*, a specialist that was collected only off of *Ludwigiapeploides*.

## Data resources

A table of coordinates for collection sites is given in Suppl. material 1. A spreadsheet of syrphid occurrence data reported here is in Suppl. material 2. Raw values for the syrphid pollen analysis are reported in Suppl. material 3.

## Results

### Faunal composition

The floral visitor surveys used in this study yielded a total of 33,563 insect specimens, of which 1,477 were Syrphidae (4.40% of the total collection). The rest of the collection was comprised of 60.21% bees, 5.66% non-bee Hymenopterans, 12.92% non-syrphid Diptera, 5.07% Coleoptera, and 11.74% Lepidoptera; these will be reported on elsewhere.

The 1477 syrphids represent 69 species belonging to 36 genera (Table 1). Sixty-four of these species were identified as valid described species, 1 identified as a currently-undescribed species ('*Palpada* undescribed species 1' according to Skevington et al. 2019), and 4 taxa identified to species groups or affinities. The most abundant species in the collection were *Toxomerusmarginatus* (45.63% of all collections), *Toxomerusgeminatus* (13.61%), *Paragushaemorrhous* (3.05%), *Toxomeruspolitus* (2.91%), *Milesiavirginiensis* (2.17%), *Toxomerusboscii* (2.03%), and *Eristalistransversa* (1.96%).

To our knowledge, only one species historically observed in Southern Illinois was not collected during our inventory: *Temnostomatrifasciatum* (Robertson, 1901), known from one 1951 Union County specimen held at the Smithsonian National Museum of Natural History (NMNH, catalog number USNMENT 1541967). All other syrphid records from Southern Illinois at the NMNH, Illinois Natural History Survey, and iNaturalist Research-grade Observations represent species collected in this study.

**Figure 7.**
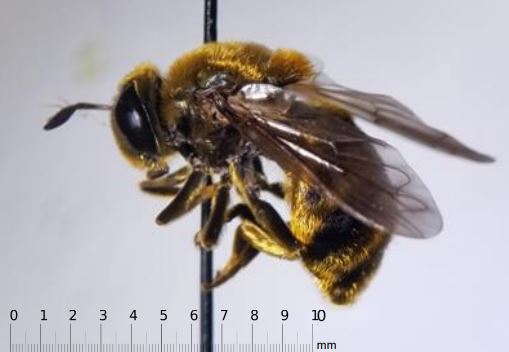
Collected specimen of *Microdonaurulentus*, with scale.

**Figure 8.**
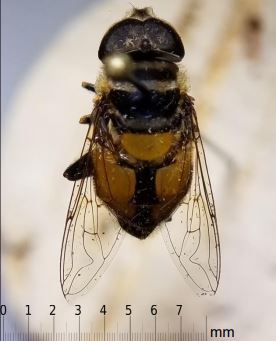
Collected specimen of *Palpadaagrorum*, with scale.

Collections of note include *Microdonaurulentus Fig. 7*, which is known only from <10 records from US States Ohio-Georgia-Pennsylvania (GBIF.org 2020c, Skevington et al. 2019). *Palpadaagrorum Fig. 8*, represented by 6 records in this collection, is a common species along the Gulf Coast and into Oklahoma but had previously not been collected as far north along the Mississippi river as Illinois. Introduced species comprised 1.9% of the total collection and include *Eristalisarbustorum* (n=1), *Eristalistenax* (n=3), *Syrittaflaviventris* (n=1), and *Syrittapipiens* (n=23).

### Species pool estimate

**Figure 9.**
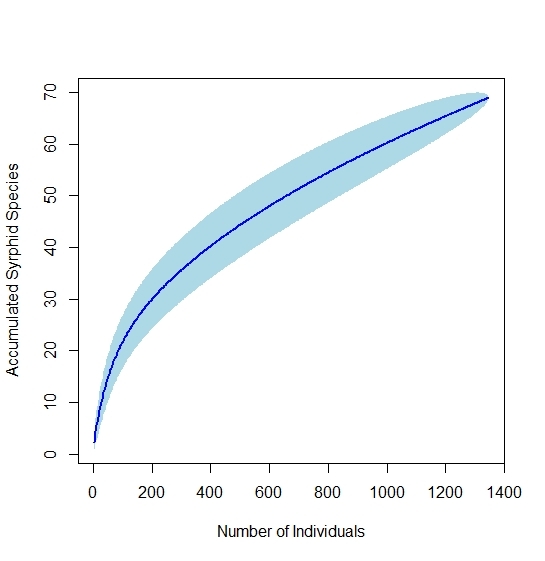
Species accumulation curve for syrphid individuals collected from 2017-2019. Light blue confidence intervals show standard deviation.

The species accumulation curve (Fig. 9) does not approach asymptotic, suggesting that surveying was not adequate to capture the full syrphid species richness of Southern Illinois. First-order jackknife estimates the total species pool to be 101.93 (standard error 6.07). Bootstrap estimate predicted a lower number, 82.47 species (standard error 3.12). These values may underestimate the real regiona richness, as discussed below.

### Floral associations

Of the 1477 syrphid specimens collected, 1047 (70.89%) were collected by hand-netting off of flowers and 107 (7.24%) were collected by hand-netting while flying. Syrphids were collected from the flowers of 157 plant species representing 47 plant families in Table 2. Collections from flowers yielded 62 syrphid species, 41 of which were never collected in pan traps. Of syrphids collected off flowers, Asteraceae comprised 50.78% of collections, followed by Fabaceae (7.39%), Apiaceae (6.23%), Oxalidaceae (3.79%), Brassicaceae (3.70%), Ranunculaceae (2.82%), and 41 other plant families (the remaining 25.29%). Pan trapping collected 323 (21.87%) syrphids, constituting 28 species. Seven species were collected in pan traps but never collected in nets: *Chalcosyrphus libo, Chalcosyrphus nemorum, Microdon manitobensis, Teuchocnemisbacuntius, Teuchocnemislituratus, Trichopsomyiaapisaon*, and *Xylotaejuncida.* Each of these species was represented by just one individual.

### Floral association NMDS

**Figure 10.**
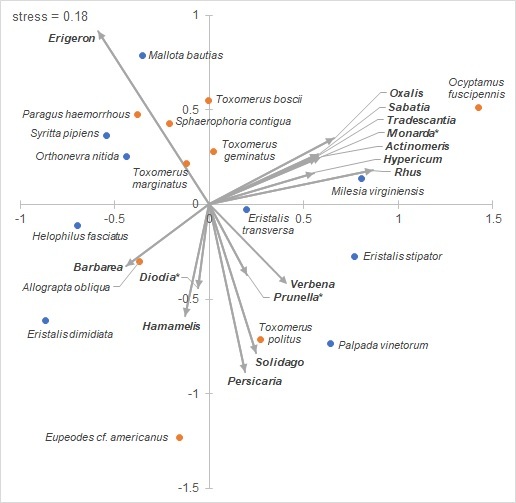
NMDS ordination of 18 syrphid species by floral association genera. Blue points represent species in subfamily Eristalinae, orange points Syrphinae. Significant (alpha=0.1) plant vectors are shown. Plant genera names followed by an asterisk have p<0.1; all others have p<0.05.

The NMDS of floral associations is given in Fig. 10.

### Pollen load comparison

**Figure 11.**
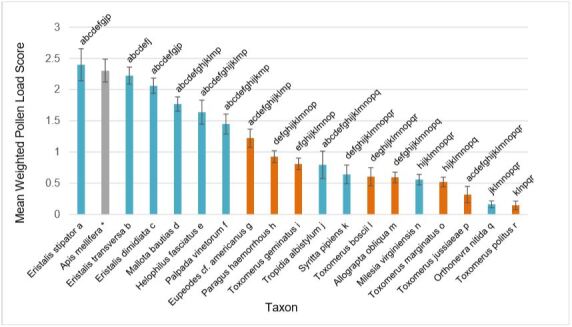
Mean weighted pollen scores for each species analyzed, with standard error bars. Blue bars represent species in subfamily Eristalinae, orange bars Syrphinae, and grey bar *Apismellifera*. Shared letters above bars denote no significant pairwise difference (pairwise Wilcoxon Rank Sum Test, alpha=0.05 with Bonferroni correction). *Apismellifera* is included for comparison to syrphids but was not included in pairwise tests.

Pollen scores for each of the 18 species analyzed are summarized in Fig. 11. Species ranged from mean scores of 2.33 (*Eristalisstipator*) to 0.15 (*Toxomeruspolitus*).

## Discussion

The results of this inventory have provided a baseline of Syrphidae species richness and relative abundance in the Southern Illinois region. The genus *Toxomerus* represents the majority of the flower-visiting syrphids, comprising 69% of all syrphid individuals collected. Our collections near agricultural areas (Crab Orchard National Wildlife Refuge, Sparta National Guard Training Area) likely contribute to the abundance of the three most commonly collected species (*Toxomerusmarginatus*, *T.geminatus*, and *Paragushaemorrhous*), as larvae of these species are common predators of crop pests (Eckberg et al. 2014), especially aphids. While the subfamily Syrphinae outnumbered Eristalinae in abundance by a factor of 4.4, species richness in the Syrphinae (23 species) was only 59% that of the Eristalinae (39 species).

Though sampling for this inventory was thorough (756 collection events), the species accumulation curve (Fig. 9) suggests that sampling failed to capture much of the regional syrphid diversity; the curve rises at a nearly constant slope after the 400th individual, rather than leveling off as expected if the full regional richness was captured. The first-order jackknife estimate (102 species) suggests that as little as 68% of the regional species pool may be known. This is, however, very likely an underestimation, as the jackknife estimates the species pool of the sites, rather than the region as a whole. Additionally, some habitats may have been undersampled, wetlands, which generally contain high syrphid diversity. The first-order jackknife estimation of 102 species may be used as a lower limit for the regional species pool, though more sampling will be required to fully document the syrphid richness of the Southern Illinois region. While collections for this study encompassed a broad range of floral visitors, collection targeting syrphids would be more productive; malaise traps should be employed, which have been shown to be efficient in capturing syrphid diversity (Burgio and Sommaggio 2007). One group likely to be undersampled by our methodology is the genus *Microdon*, a group of ant nest predators which do not regularly associate with flowers (Duffield 1981). The single record of *Microdonglobosus* visiting *Medicagolupulina* is of note, as there are very few observations of Microdontinae visiting flowers (M. Reemer, personal communication). Just four *Microdon* specimens were collected (3 in pan traps), constituting four different species. As *Microdon* are not typically flower visitors, however, they are unlikely to be pollinators of any import in our region.

At 69 species, Syrphidae is one of the most diverse groups of floral visitors collected in our 2017-2019 surveys. Bee families yielded from 19 (Colletidae) to 67 (Apidae) species, and butterflies including skippers (Lepidoptera: Rhopalocera) yielded 72 species. One reason for the high syrphid richness documented may be that Southern Illinois is predominantly rural; syrphid abundance and richness have both been shown to decline with increasing urbanization (Udy et al. 2020). Seventeen species (25% of total) reported in this study have not been recorded in Illinois before (Table 1), according to records in Skevington et al. (2019) and 10 datasets in GBIF.org (2020d). This demonstrates the large gap in our knowledge of syrphid distribution in the Eastern US, stressing the need for further studies of this diverse group of pollinators.

Syrphids were collected from a wide range of flowers (157 species). Floral associations generally followed the predicted pattern for non-carrion fly pollination syndromes: white, yellow, green, or brown flowers in color, radial symmetry, exposed pollen and nectar (Faegri and Pijl 1979). Over half of flower visits observed were to Asteraceae (40 floral species, 28 syrphid species collected from). The 25 most common floral associations (all those with more than 7 syrphids collected) have either white perianths, yellow perianths, or both (as in the bicolored capitula of *Erigeron* and *Leucanthemum*) except for *Trifoliumpratense* (pink flowers) and *Plantagolanceolata* (anemophilous without showy flowers, though anthers are large and white). However, the pink flowers of *T.pratense* may not differ visually from white flowers to syrphids, as flies exhibit low sensitivity to red light (Lunau 2014). The frequency of Fabaceous flowers as floral associations (7.39% of the total; second most commonly visited plant family) is of note as the Fabaceous flowers collected off of (mostly *Trifolium*) possess tubular rather than open corollae, contrasting with the classical fly pollination syndrome. However, pollination syndromes have been shown to be poor predictors of floral visitation (Ollerton et al. 2009), and syrphids have been documented to forage on *Trifolium* species (Larson et al. 2014) and are likely pollinators.

The NMDS of floral associations failed to sort syrphid species into discrete guilds (Fig. 10), though some clustering is apparent. Syrphinae species are all (except for *Ocyptamusfuscipennis*) within ±0.5 of axis 1, whereas Eristalinae is far more evenly distributed in the ordination space. Eristalinae does cluster into two long groups on either side of axis 1, though this grouping is loose and does not reflect strong similarity in floral association within the Eristalinae. Five small predatory Syrphines (*Paragushaemorrhous*, *Sphaerophoriacontigua*, *Toxomerusboscii*, *T.geminatus*, *T.marginatus*) and two small Eristalines (*Orthonevranittida*, *Syrittapipiens*) are grouped around the *Erigeron* vector, the strongest vector in the ordination (p<0.001). Each of these species are common and exhibit low (below 1 mean pollen load score) pollen-carrying ability (Fig. 11). This group may act as abundant but low-quality pollinators of *Erigeron* and other weedy plant species. *Toxomerus* species except for *T.politus* are grouped in ordination space, showing high similarity in floral visitation within the genus. Larvae of *T.politus* feed on pollen of corn (*Zeamays*) (Reemer and Rotheray 2009), whereas other *Toxomerus* species in our area are predatory (*T.marginatus*, *T.geminatus*, *T.boscii*) or unknown (*T.jussiaeae*) Skevington et al. 2019; this difference in life history may play a role in the different floral visitation patterns exhibited by *T.politus* and its congenerics. *T.politus* also carries less pollen than other *Toxomerus* species (Fig. 11), which does not support grouping the whole genus as an ecologically similar guild. The grouping of *Toxomerus* (except for *T.politus*) in ordination space is in contrast to *Eristalis*, the three species of which are widely separated in the NMDS. *Milesiavirginiensis* and *Ocyptamusfuscipennis* are at the most positive values of axis 1; both species inhabit forests (Skevington et al. 2019), which is reflected by the vectors in their quadrant of the ordination (forest plants such as *Tradescantia* and *Actinomeris*).

Examination of pollen loads showed significant differences in pollen carrying capacity of syrphid species (Fig. 11). Of note, the pollen analysis scored pollen coverage rather than number of pollen grains. Pollen coverage may be more important than pollen count in successful pollination, though we are aware of no studies assessing this. Many pairwise comparisons were not significant likely due to low sample sizes. Even so, some trends are clear. The six syrphids with the highest pollen scores were all in the tribe Eristalini of the Eristalinae, large bodied, and pilose: *Eristalisstipator*, *E.transversa*, *E.dimidiata*, *Mallotabautias*, *Helophilusfasciatus*, and *Palpadavinetorum*. This is expected, as pilosity and size are positively correlated with pollen load in flies (Inouye et al. 2015). This generalization is not a rule, however; *Milesiavirginiensis* is large and pilose yet scored in the bottom five of the 18 species analyzed. The three *Eristalis* species analyzed all scored within ±0.25 of *Apismellifera*, with *Eristalisdimidiata* even scoring slightly above. The high pollen load scores of these Eristalines has definite implications for the quality of the species as pollinators. Still, other factors such as floral constancy and pollen deposition on stigmas need be considered in order to further quantify their efficacy (Herrera 1987).

Several syrphids were collected in February, extremely early for floral visitors in the region: Eupeodescf.americanus, *Eristalisdimidiata*, and *Syrphustorvus*. These specimens were collected off of a cultivated *Hamamelisvirginiana* (American witch-hazel) on the SIUC campus. Considering the high pollen scores of *Eristalisdimidiata* and *Eupeodes*, these species may be important pollinators in the very early spring, before bees and most other floral visitors are flying.

The high pollen scores of the tribe Eristalini contrast greatly with many of the Syrphinae and less pilose Eristalinae. *Orthonevranitida* and *Toxomeruspolitus* carried almost no pollen, and are thus unlikely to pollinate with any consistency. *Toxomerusmarginatus* and *T.boscii* each scored ~0.5 on average, frequently carrying no pollen at all. *T.geminatus* scored slightly higher, though pairwise tests between the *Toxomerus* species were not significant. This is of note because *Toxomerus* was the most abundant genus of syrphids by far (69% of total). The similarity in pollen load size and floral association (Fig. 10) suggests that *Toxomerusmarginatus*, *T.geminatus*, and *T.boscii* may be treated as a guild of similar pollinators. While their abundance may compensate for their low quality, consumption of floral resources by *Toxomerus* without pollen deposition on stigmas may harm plant reproduction. In contrast, *Eristalis* spp and other large pilose Eristalini syrphids are likely to be important pollinators where they occur, though their relatively low abundance means that these species are not ubiquitous across the Southern Illinois landscape and their importance as pollinators will be localized to where they are abundant.

## Supplementary Material

A705CAAA-B6EF-5CD0-A1C5-BC88947B415E 10.3897/BDJ.8.e57331.suppl1 Supplementary material 1 Table of localities Data type Coordinates Brief description Matrix of coordinates of all sites from which syrphids were collected File: oo_451714.xlsx https://binary.pensoft.net/file/451714 Jacob Chisausky

299E1265-D7C0-5F20-A8E1-D62BA3E7FFBA 10.3897/BDJ.8.e57331.suppl2 Supplementary material 2 Collection occurrence data Data type Occurrence Brief description Data matrix of 1477 syrphids collected in Southern Illinois from 2017-2019, including species determinations, locality data, and floral associations. File: oo_451713.xlsx https://binary.pensoft.net/file/451713 Jacob Chisausky

DD85AFF3-9CED-556F-BE09-56E1CA6A8D1C 10.3897/BDJ.8.e57331.suppl3 Supplementary material 3 Syrphid pollen analysis data Data type Pollen load scores Brief description Data matrix from analysis of pollen load size of 416 syrphid specimens. Pollen coverage for eight regions of the body was assigned a score of 0-5 for each specimen. File: oo_404050.xlsx https://binary.pensoft.net/file/404050 Jacob Chisausky

## Figures and Tables

**Table 1.** List of syrphid species collected in Randolph county and the southernmost 11 counties of Illinois, United States, from 2017-2019 with number and date range of specimens collected. Floral associations are reported for each species and correspond to family and species codes given in Table 2. Taxa with no floral associations listed were collected only from pan traps and/or free flying. Full occurrence records are provided in Suppl. material 2. ^†^This species not previously documented from Illinois. Compared to occurrence records in Skevington et al. (2019), GBIF.org (2020d) ^1^This species previously known only from Virginia, US-New Brunswick, Canada (Skevington et al. 2019). ^2^Introduced species from Palearctic. ^3^This species previously known only from US States Oklahoma-North Carolina, south to Argentina (GBIF.org 2020b). ^4^Undescribed species closely related to *P.furcata* (Skevington et al. 2019). ^5^This species previously only known from <10 records from US States Ohio-Georgia-Pennsylvania (GBIF.org 2020c, Skevington et al. 2019). ^6^*E.americanus* or *pomus*.

**Taxonomic name (Author, Year)**	# **of Specimens**	**Months Collected**	**Floral Associations**
**Family Syrphidae**	**1477**	Feb-Sep	
**Subfamily Eristalinae**	**257**	Feb-Sep	
Chalcosyrphus (Xylotomima) libo (Walker, 1849) ^†^	1	Apr	
Chalcosyrphus (Xylotomima) metallicus (Wiedemann, 1830)	5	Jun-Aug	**Adox**: *Samnig***Aste**: *Eristr***Rubi**: *Cepocc*
Chalcosyrphus (Xylotomima) nemorum (Fabricius, 1805)	1	Apr	
Cheilosiaaff.florella	1	Apr	**Ranu**: *Ran*
Cheilosiaaff.platycera	1	Apr	
*Cheilosiaprimoveris* (Shannon, 1915) ^†,1^	6	Mar	**Port**: *Clavir*
*Cheilosiawisconsinensis* (Fluke & Hull, 1947) ^†^	3	Mar-Jul	**Anac**: *Rhucop***Aste**: *Vervir*
Copestylum (Phalacromya) vesicularium (Curran, 1947)	3	May-Aug	**Aste**: *Acthel***Ranu**: *Anevir*
Eristalis (Eoseristalis) arbustorum (Linnaeus, 1758) ^2^	1	Jun	**Apia**: *Daucar*
Eristalis (Eoseristalis) dimidiata (Wiedemann, 1830)	12	Feb-Sep	**Aste**: *Bolast, Eri, Sengla***Bras**: *Barvul***Hama**: *Hamvir***Rosa**: *Pyrcal*
Eristalis (Eoseristalis) flavipes (Walker, 1849)	3	May-Jul	**Apia**: *Daucar***Faba**: *Tripra*
Eristalis (Eoseristalis) stipator (Osten Sacken, 1877)	18	May-Aug	**Aste**: *Cirvul, Cor, Eristr, Helhel, Leuvul, Rudhir, Vermis***Lami**: *Menpip***Ranu**: *Ranabo***Verb**: *Phylan, Verhas*
Eristalis (Eoseristalis) transversa (Wiedemann, 1830)	29	Apr-Sep	**Apia**: *Daucar***Aste**: *Acthel, Bid, Cor, Eriann, Hel, Helhel, Leuvul, Rudhir, Rudsul, Sengla***Eric**: *Vacarb*
Eristalis (Eristalis) tenax (Linnaeus, 1758) ^2^	3	Feb-Jun	**Aste**: *Eri***Eric**: *Vacarb***Hama**: *Hamvir*
Helophilus (Helophilus) fasciatus (Walker, 1849)	25	Apr-May	**Apia**: *Chatai, Daucar***Aste**: *Eriphi, Kri, Sengla***Bora**: *Phapur***Bras**: *Barvul***Cary**: *Stemed***Corn**: *Corfoe***Faba**: *Trirep***Lami**: *Blehir***Papa**: *Stydip*
Mallota (Mallota) bautias (Walker, 1849)	8	Apr-Jun	**Adox**: *Samnig***Aste**: *Eriphi, Eristr***Bras**: *Brarap***Hydr**: *Hydarb*
Mallota (Mallota) posticata (Fabricius, 1805)	1	Jun	**Ranu**: *Anevir*
*Milesiavirginiensis* (Drury, 1773)	32	Jun-Sep	**Anac**: *Rhugla***Aste**: *Ech, Eri, Hel, Liapyc, Rudhir, Symeri***Faba**: *Trirep***Hype**: *Hyppro***Oxal**: *Oxastr***Rubi**: *Cepocc*
Myolepta (Myolepta) pretiosa (Hull, 1923) ^†^	1	Apr	**Corn**: *Corflo*
Myolepta (Myolepta) strigilata (Loew, 1872)	2	Apr	**Rosa**: *Prupad*
*Orthonevranitida* (Wiedemann, 1830)	17	Apr-Jul	**Adox**: *Samnig***Apia**: *Daucar*, *Eryyuc***Aste**: *Achmil, Eristr, Leuvul***Bras**: *Barvul***Faba**: *Melalb***Lami**: *Pycten*
*Palpadaagrorum* (Fabricius, 1787) ^†,3^	6	Jun-Jul	**Apia**: *Daucar***Aste**: *Cirvul*, *Eristr***Faba**: *Trirep*
*Palpada* undescribed species 1 ^†,4^	1	Jul	**Apia**: *Daucar*
*Palpadavinetorum* (Fabricius, 1799)	27	Jun-Sep	**Anac**: *Rhu***Apia**: *Daucar, Eryyuc, Torarv***Aste**: *Elecar, Eristr, Eupser, Heldiv, Rudhir, Solalt, Soljun***Capr**: *Symorb***Dips**: *Dipful***Faba**: *Melalb***Lami**: *Pruvul*, *Pycten***Poly**: *Per***Verb**: *Verhas, Verurt*
*Parhelophilusinteger* (Loew, 1863) ^†^	1	Apr-Apr	**Papa**: *Stydip*
*Parhelophiluslaetus* (Loew, 1863)	1	May	**Corn**: *Corfoe*
*Pterallastesthoracicus* (Loew, 1863)	3	Jun-Jul	**Adox**: *Samnig***Camp**: *Camame*
*Sphecomyiavittata* (Wiedemann, 1830)	1	Apr	**Bora**: *Mervir*
Sphegina (Asiosphegina) petiolata (Coquillett, 1910) ^†^	1	May	**Corn**: *Corfoe*
*Spilomyiaalcimus* (Walker, 1849)	2	Jun-Jun	**Adox**: *Samnig*
*Spilomyialongicornis* (Loew, 1872)	3	Jul-Sep	**Aste**: *Bolast*, *Eup*, *Soljun*
*Syrittaflaviventris* (Macquart, 1842) ^†,2^	1	Jul	**Verb**: *Verurt*
*Syrittapipiens* (Linnaeus, 1758) ^2^	23	May-Aug	**Apia**: *Daucar*, *Torarv***Aste**: *Cicint*, *Eristr*, *Olirig*
*Temnostomabalyras* (Walker, 1849) ^†^	2	Apr-May	**Faba**: *Medlup*
*Temnostomadaochus* (Walker, 1849)	1	Apr	**Corn**: *Corflo*
*Teuchocnemisbacuntius* (Walker, 1849)	1	Apr	
*Teuchocnemislituratus* (Loew, 1863)	1	Apr	
Tropidia (Tropidia) albistylum (Macquart, 1847)	8	May-Jul	**Apia**: *Chapro***Aste**: *Eriphi*, *Eristr***Gera**: *Gercar***Poly**: *Per***Rubi**: *Diovir*
Xylota (Xylota) ejuncida (Say, 1824) ^†^	1	Sep	
**Subfamily Microdontinae**	**4**	May-Jun	
Microdon (Dimeraspis) abditus (Thompson, 1981)	1	May	
Microdon (Dimeraspis) globosus (Fabricius, 1805) ^†^	1	Jun	**Faba**: *Medlup*
Microdon (Microdon) aurulentus (Fabricius, 1805) ^†,5^	1	May	
Microdon (Microdon) manitobensis (Curran, 1924) ^†^	1	May	
**Subfamily Pipizinae**	**9**	Apr-Aug	
Heringia (Heringia) salax (Loew, 1866)	3	May-Aug	**Camp**: *Camame***Oxal**: *Oxastr*
*Pipizafemoralis* (Loew, 1866)	5	Apr-Apr	**Port**: *Clavir***Scro**: *Colver***Viol**: *Viosor*
*Trichopsomyiaapisaon* (Walker, 1849)	1	Apr	
**Subfamily Syrphinae**	**1122**	Feb-Sep	
Allograpta (Allograpta) exotica (Wiedemann, 1830) ^†^	1	May	**Faba**: *Medlup*
Allograpta (Allograpta) obliqua (Say, 1823)	18	Apr-Jul	**Apia**: *Conmac*, *Daucar*, *Torarv***Aste**: *Eri*, *Sengla***Faba**: *Cercan***Hypo**: *Hyphir***Oxal**: *Oxastr***Ranu**: *Ran*
*Epistrophellaemarginata* (Say, 1823)	2	Jun-Aug	**Aste**: *Vermis***Lami**: *Monfis*
Eupeodescf.americanus ^6^	24	Feb-Sep	**Aste**: *Bidpol*, *Heldiv, Kri, Sengla, Solcan***Bora**: *Bugarv***Bras**: *Barvul***Capr**: *Valloc***Hama**: *Hamvir***Hype**: *Hyp***Poly**: *Per***Rubi**: *Diovir*
*Eupeodeslatifasciatus* (Macquart, 1829) ^†^	1	Apr	**Bras**: *Lepvir*
*Ocyptamusfascipennis* (Wiedemann, 1830)	1	Jun	**Bras**: *Lepvir*
*Ocyptamusfuscipennis* (Say, 1823)	10	Jun-Jul	**Anac**: *Rhugla***Aste**: *Acthel***Comm**: *Trad***Gent**: *Sabang***Hype**: *Hyp***Lami**: *Monfis***Oxal**: *Oxastr*
Paragus (Pandasyopthalmus) haemorrhous (Meigen, 1822)	45	Apr-Sep	**Apia**: *Daucar***Aste**: *Ant, Eriann, Eristr, Eup, Helpau***Bras**: *Bra***Euph**: *Eupcor***Lami**: *Teucan***Plan**: *Plalan***Rubi**: *Hou*
Paragus (Paragus) angustifrons (Loew, 1863)	1	Aug	**Aste**: *Elecar*
*Pelecinobacchacostata* (Say, 1829)	2	Jun	**Anac**: *Rhugla***Faba**: *Medlup*
Platycheiruscf.albimanus	1	May	
*Pseudodorosclavatus* (Fabricius, 1794)	4	Jul-Aug	**Verb**: *Verhas*, *Verurt*
*Sphaerophoriacontigua* (Macquart, 1847)	23	Apr-Jun	**Apoc**: *Apocan***Aste**: *Eristr, Leuvul, Sengla***Bras**: *Lepvir***Capr**: *Valloc***Oxal**: *Oxastr***Ranu**: *Ranbul*
*Syrphusknabi* (Shannon, 1916)	1	May	
*Syrphusrectus* (Osten Sacken, 1875)	1	May	
*Syrphusribesii* (Linnaeus, 1758)	1	Mar	**Lami**: *Pyc*
*Syrphustorvus* (Osten Sacken, 1875)	1	Feb	**Hama**: *Hamvir*
*Toxomerusboscii* (Macquart, 1842) ^†^	30	Apr-Sep	**Alis**: *Alisub***Aste**: *Eriann, Eristr, Leuvul***Cary**: Cerglo **Faba**: *Medlup, Trirep***Hype**: *Hypdru***Oxal**: *Oxastr***Ranu**: *Ranabo, Ranbul, Ranpus***Verb**: *Phylan*
*Toxomerusgeminatus* (Say, 1823)	201	Mar-Sep	**Apia**: *Daucar, Osmcla, Torarv***Aste**: *Acthel, Cicint, Eriann, Eriphi, Eristr, Eup, Heldiv, Hiegro, Kri, Leuvul, Rudser, Sengla, Silint, Taroff, Vervir***Bora**: *Mervir***Bras**: *Carcon***Camp**: *Camame*, *Trilep***Capr**: *Valloc***Cary**: *Cerglo, Stemed***Comm**: *Comcom, Tradvir***Corn**: *Corfoe***Cras**: *Sedpul***Euph**: *Eupcor***Faba**: *Medlup, Melalb, Secvar, Trirep***Gent**: *Sabang***Hydr**: *Hydarb***Lami**: *Blehir, Lampur, Pruvul, Pyc***Lyth**: *Ludalt***Oxal**: *Oxastr***Phry**: *Mimala***Plan**: *Penhir***Pole**: *Phlpil, Polrep***Port**: *Clavir***Rosa**: *Geucan***Rubi**: *Galapa***Verb**: *Phrlep*
*Toxomerusjussiaeae* (Vige, 1939)	9	Jul-Aug	**Lyth**: *Ludpep*
*Toxomerusmarginatus* (Say, 1823)	674	Apr-Sep	**Adox**: *Samnig***Alis**: *Alisub***Apia**: *Conmac, Daucar, Osmcla, Taeint, Torarv***Aste**: *Achmil, Acthel, Cirvul, Concan, Cor, Eriann, Eriphi, Eristr, Eup, Kri, Kribif, Leuvul, Parint, Rudhir, Rudtri, Sengla, Sympil, Taroff***Bora**: *Phapur***Bras**: *Barvul, Bra, Lepvir, Rorten***Camp**: *Trilep, Triper***Capr**: *Valloc***Cary**: *Cerglo, Cervul, Diaarm, Stemed***Euph**: *Cromon***Faba**: *Lotcor, Medlup, Medsat, Melalb, Meloff, Secvar, Triinc, Tripra, Trirep, Vicvil***Gera**: *Gercar***Hypo**: *Hyphir***Irid**: *Sisang***Lami**: *Pruvul***Oxal**: *Oxastr***Plan**: *Pendea, Pendig, Plalan, Verarv, Verper***Poly**: *Per***Port**: *Clavir***Ranu**: *Deltri, Ranabo, Ranbul, Ransar***Rosa**: *Amecan, Pot, Pyrcal***Rubi**: *Cepocc, Diovir, Houlon***Sola**: *Solcar***Verb**: *Phylan*
*Toxomeruspolitus* (Say, 1823)	43	Jul-Aug	**Acan**: *Ruehum***Apia**: *Daucar***Aste**: *Eri, Eutfis, Hel, Rud, Sol***Camp**: *Camame***Conv**: *Ipolac***Faba**: *Tripra***Hydr**: *Hydarb***Lami**: *Pruvul*, *Sta***Malv**: *Hiblae, Sidspi***Phry**: *Mimala***Poac**: *Zeamay***Poly**: *Per***Verb**: *Verhas*
*Xanthogrammaflavipes* (Loew, 1863)	1	Jun	**Amar**: *Allcan*
**Unidentified to species**	**85**	-	

**Table 2.** List of all floral taxa from which syrphids were collected. Plant species codes (as reported in Table 1) are comprised of the first three letters of the genus and specific epithet, and family codes are comprised of the first four letters of the family name. Floral associations were occasionally identified only to genus level, and these are reported in Table 1 as the first three letters of the genus name (except *Tradescantia* and *Tragopogon*, for which the first four letters are used).

Taxon	Taxon Code	# syrphid specimens collected from	# syrphid species collected from
Pan Trap		323	28
**Acanthaceae**	**Acan**	**1**	**1**
*Ruelliahumilis*	*Ruehum*	1	1
**Adoxaceae**	**Adox**	**11**	**6**
*Sambucusnigra*	*Samnig*	11	6
**Alismataceae**	**Alis**	**9**	**2**
*Alismasubcordatum*	*Alisub*	9	2
**Amaryllidaceae**	**Amar**	**1**	**1**
*Alliumcanadense*	*Allcan*	1	1
**Anacardiaceae**	**Anac**	**5**	**5**
*Rhuscopallinum*	*Rhucop*	1	1
*Rhusglabra*	*Rhugla*	3	3
*Rhusspp*	*Rhu*	1	1
**Apiaceae**	**Apia**	**61**	**15**
*Chaerophyllumprocumbens*	*Chapro*	1	1
*Chaerophyllumtainturieri*	*Chatai*	1	1
*Coniummaculatum*	*Conmac*	3	2
*Daucuscarota*	*Daucar*	41	15
*Eryngiumyuccifolium*	*Eryyuc*	3	2
*Osmorhizaclaytonii*	*Osmcla*	4	2
*Taenidiaintegerrima*	*Taeint*	1	1
*Torilisarvensis*	*Torarv*	7	5
**Apocynaceae**	**Apoc**	**1**	**1**
*Apocynumcannabinum*	*Apocan*	1	1
**Asteraceae**	**Aste**	**521**	**28**
*Achilleamillefolium*	*Achmil*	4	2
*Actinomerishelianthoides*	*Acthel*	5	5
*Antennaria*spp.	*Ant*	1	1
*Bidenspolylepis*	*Bidpol*	1	1
*Bidens*spp.	*Bid*	2	1
*Boltoniaasteroides*	*Bolast*	2	2
*Cichoriumintybus*	*Cicint*	2	2
*Cirsiumvulgare*	*Cirvul*	3	3
*Conyzacanadensis*	*Concan*	1	1
*Coreopsis*spp.	*Cor*	4	3
*Echinacea*spp.	*Ech*	1	1
*Elephantopuscarolinianus*	*Elecar*	2	2
*Erigeronannuus*	*Eriann*	27	5
*Erigeronphiladelphicus*	*Eriphi*	26	5
*Erigeron*spp.	*Eri*	139	15
*Erigeronstrigosus*	*Eristr*	95	14
*Eupatoriumserotinum*	*Eupser*	1	1
*Eupatorium*spp.	*Eup*	4	4
*Eutrochiumfistulosum*	*Eutfis*	1	1
*Helianthusdivaricatus*	*Heldiv*	5	3
*Helianthuspauciflorus*	*Helpau*	1	1
*Helianthus*spp.	*Hel*	10	4
*Heliopsishelianthoides*	*Helhel*	3	2
*Hieraciumgronovii*	*Hiegro*	1	1
*Krigiabiflora*	*Kribif*	1	1
*Krigia*spp.	*Kri*	44	5
*Leucanthemumvulgare*	*Leuvul*	28	8
*Liatrispycnostachya*	*Liapyc*	1	1
*Oligoneuronrigidum*	*Olirig*	1	1
*Partheniumintegrifolium*	*Parint*	1	1
*Rudbeckiahirta*	*Rudhir*	17	5
*Rudbeckiaserotina*	*Rudser*	1	1
*Rudbeckia*spp.	*Rud*	16	5
*Rudbeckiasullivantii*	*Rudsul*	1	1
*Rudbeckiatriloba*	*Rudtri*	1	1
*Senecioglabellus*	*Sengla*	51	9
*Silphiumintegrifolium*	*Silint*	2	1
*Solidagoaltissima*	*Solalt*	1	1
*Solidagocanadensis*	*Solcan*	1	1
*Solidagojuncea*	*Soljun*	2	2
*Solidago*spp.	*Sol*	2	2
*Symphyotrichumericoides*	*Symeri*	1	1
*Symphyotrichumpilosum*	*Sympil*	1	1
*Taraxacumofficinale*	*Taroff*	2	2
*Tragopogon*spp.	*Trag*	1	1
*Verbesinavirginica*	*Vervir*	2	2
*Vernoniamissurica*	*Vermis*	2	2
**Boraginaceae**	**Bora**	**5**	**5**
*Buglossoidesarvensis*	*Bugarv*	1	1
*Mertensiavirginica*	*Mervir*	2	2
*Phaceliapurshii*	*Phapur*	2	2
**Brassicaceae**	**Bras**	**38**	**11**
*Barbareavulgaris*	*Barvul*	13	5
*Brassicarapa*	*Brarap*	1	1
*Brassica*spp.	*Bra*	4	2
*Cardamineconcatenata*	*Carcon*	1	1
*Lepidiumvirginicum*	*Lepvir*	18	5
*Rorippatenerrima*	*Rorten*	1	1
**Campanulaceae**	**Camp**	**12**	**5**
*Campanulastrumamericanum*	*Camame*	6	4
*Triodanisleptocarpa*	*Trilep*	3	2
*Triodanisperfoliata*	*Triper*	3	1
**Caprifoliaceae**	**Capr**	**17**	**5**
*Symphoricarposorbiculatus*	*Symorb*	1	1
*Valerianellalocusta*	*Valloc*	16	5
**Caryophyllaceae**	**Cary**	**9**	**4**
*Cerastiumglomeratum*	*Cerglo*	3	3
*Cerastiumvulgatum*	*Cervul*	1	1
*Dianthusarmeria*	*Diaarm*	2	1
*Stellariamedia*	*Stemed*	3	3
**Commelinaceae**	**Comm**	**3**	**2**
*Commelinacommunis*	*Comcom*	1	1
*Tradescantia*spp.	*Trad*	1	1
*Tradescantiavirginiana*	*Tradvir*	1	1
**Convolvulaceae**	**Conv**	**1**	**1**
*Ipomealacunosa*	*Ipolac*	1	1
**Cornaceae**	**Corn**	**7**	**6**
*Cornusflorida*	*Corflo*	2	2
*Cornusfoemina*	*Corfoe*	5	4
**Crassulaceae**	**Cras**	**1**	**1**
*Sedumpulchellum*	*Sedpul*	1	1
**Dipsacaceae**	**Dips**	**2**	**1**
*Dipsacusfullonum*	*Dipful*	2	1
**Ericaceae**	**Eric**	**2**	**2**
*Vacciniumarboreum*	*Vacarb*	2	2
**Euphorbiaceae**	**Euph**	**9**	**3**
*Crotonmonanthogynus*	*Cromon*	1	1
*Euphorbiacorollata*	*Eupcor*	8	2
**Fabaceae**	**Faba**	**75**	**15**
*Cerciscanadensis*	*Cercan*	2	1
*Lotuscorniculatus*	*Lotcor*	2	1
*Medicagolupulina*	*Medlup*	16	7
*Medicagosativa*	*Medsat*	1	1
*Melilotusalbus*	*Melalb*	7	4
*Melilotusofficinalis*	*Meloff*	1	1
*Securigeravaria*	*Secvar*	2	2
*Trifoliumincarnatum*	*Triinc*	1	1
*Trifoliumpratense*	*Tripra*	10	3
*Trifoliumrepens*	*Trirep*	31	6
*Viciavillosa*	*Vicvil*	2	1
**Gentianaceae**	**Gent**	**3**	**2**
*Sabatiaangularis*	*Sabang*	3	2
**Geraniaceae**	**Gera**	**5**	**2**
*Geraniumcarolinianum*	*Gercar*	5	2
**Hamamelidaceae**	**Hama**	**13**	**4**
*Hamamelisvirginiana*	*Hamvir*	13	4
**Hydrangeaceae**	**Hydr**	**4**	**3**
*Hydrangeaarborescens*	*Hydarb*	4	3
**Hypericaceae**	**Hype**	**11**	**4**
*Hypericumdrummondii*	*Hypdru*	1	1
*Hypericumprolificum*	*Hyppro*	1	1
*Hypericum*spp.	*Hyp*	9	4
**Hypoxidaceae**	**Hypo**	**2**	**2**
*Hypoxishirsuta*	*Hyphir*	2	2
**Iridaceae**	**Irid**	**5**	**1**
*Sisyrinchiumangustifolium*	*Sisang*	5	1
**Lamiaceae**	**Lami**	**21**	**11**
*Blephiliahirsuta*	*Blehir*	2	2
*Lamiumpurpureum*	*Lampur*	1	1
*Menthapiperita*	*Menpip*	2	1
*Monardafistulosa*	*Monfis*	2	2
*Prunellavulgaris*	*Pruvul*	4	4
*Pycnanthemum*spp.	*Pyc*	2	2
*Pycnanthemumtenuifolium*	*Pycten*	6	2
*Stachys*spp.	*Sta*	1	1
*Teucriumcanadense*	*Teucan*	1	1
**Lythraceae**	**Lyth**	**10**	**2**
*Ludwigiaalternifolia*	*Ludalt*	1	1
*Ludwigiapeploides*	*Ludpep*	9	1
**Malvaceae**	**Malv**	**2**	**1**
*Hibiscuslaevis*	*Hiblae*	1	1
*Sidaspinosa*	*Sidspi*	1	1
**Oxalidaceae**	**Oxal**	**39**	**8**
*Oxalisstricta*	*Oxastr*	39	9
**Papaveraceae**	**Papa**	**3**	**2**
*Stylophorumdiphyllum*	*Stydip*	3	2
**Phrymaceae**	**Phry**	**2**	**2**
*Mimulusalatus*	*Mimala*	2	2
**Plantaginaceae**	**Plan**	**17**	**3**
*Penstemondeamii*	*Pendea*	1	1
*Penstemondigitalis*	*Pendig*	3	1
*Penstemonhirsuta*	*Penhir*	1	1
*Plantagolanceolata*	*Plalan*	9	2
*Veronicaarvensis*	*Verarv*	2	1
*Veronicaperegrina*	*Verper*	1	1
**Poaceae**	**Poac**	**1**	**1**
*Zeamays*	*Zeamay*	1	1
**Polemoniaceae**	**Pole**	**2**	**1**
*Phloxpilosa*	*Phlpil*	1	1
*Polemoniumreptans*	*Polrep*	1	1
**Polygonaceae**	**Poly**	**5**	**5**
*Persicaria*spp.	*Per*	5	5
**Portulacaceae**	**Port**	**7**	**4**
*Claytoniavirginica*	*Clavir*	7	4
**Ranunculaceae**	**Ranu**	**29**	**8**
*Anemonevirginiana*	*Anevir*	2	2
*Delphiniumtricorne*	*Deltri*	1	1
*Ranunculusabortivus*	*Ranabo*	9	3
*Ranunculusbulbosus*	*Ranbul*	5	3
*Ranunculuspusillus*	*Ranpus*	1	1
*Ranunculussardous*	*Ransar*	2	1
*Ranunculus*spp.	*Ran*	9	3
**Rosaceae**	**Rosa**	**9**	**4**
*Amelanchiercanadensis*	*Amecan*	1	1
*Geumcanadense*	*Geucan*	1	1
*Potentilla*spp.	*Pot*	2	1
*Prunuspadus*	*Prupad*	2	1
*Pyruscalleryana*	*Pyrcal*	3	2
**Rubiaceae**	**Rubi**	**19**	**7**
*Cephalanthusoccidentalis*	*Cepocc*	6	3
*Diodiavirginiana*	*Diovir*	7	3
*Galiumaparine*	*Galapa*	1	1
*Houstonialongifolia*	*Houlon*	3	1
*Houstonia*spp.	*Hou*	2	2
**Scrophulariaceae**	**Scro**	**1**	**1**
*Collinsiaverna*	*Colver*	1	1
**Solanaceae**	**Sola**	**1**	**1**
*Solanumcarolinense*	*Solcar*	1	1
**Verbenaceae**	**Verb**	**17**	**8**
*Phrymaleptostachya*	*Phrlep*	1	1
*Phylalanceolata*	*Phylan*	7	3
*Verbenahastata*	*Verhas*	6	4
*Verbenaurticifolia*	*Verurt*	3	3
**Violaceae**	**Viol**	**1**	**1**
*Violasororia*	*Viosor*	1	1

## References

[B5804874] Baum Kristen A., Wallen Kenneth E. (2011). Potential bias in pan trapping as a function of floral abundance. Journal of the Kansas Entomological Society.

[B5818666] Burgio G., Sommaggio D. (2007). Syrphids as landscape bioindicators in Italian agroecosystems. Agriculture, Ecosystems & Environment.

[B5830715] Droege Sam, Engler Joseph D., Sellers Elizabeth A., O'Brien Lee (2016). National protocol framework for the inventory and monitoring of bees.

[B5822696] Duffield R. M (1981). Biology of *Microdonfuscipennis* (Diptera, Syrphidae) with interpretations of the reproductive strategies Of Microdon Species Found North Of Mexico. Proceedings of the Entomological Society of Washington.

[B5818553] Eckberg J. O., Peterson J. A., Borsh C. P., Kaser J. M., Johnson G. A., Luhman J. C., Wyse D. L., Heimpel G. E. (2014). Field abundance and performance of hoverflies (Diptera: Syrphidae) on Soybean Aphid. Annals of the Entomological Society of America.

[B5781979] Erhardt Andreas (2008). Pollination of the edelweiss, *Leontopodiumalpinum*. Botanical Journal of the Linnean Society.

[B5822765] Faegri K., Pijl L. van der (1979). The Principles of Pollination Ecology.

[B5983158] Fattorini Simone (2013). Regional insect inventories require long time, extensive spatial sampling and good will. PLoS ONE.

[B5781104] Forup Mikael Lytzau, Henson Kate S. E., Craze Paul G., Memmott Jane (2007). The restoration of ecological interactions: plant-pollinator networks on ancient and restored heathlands. Journal of Applied Ecology.

[B5782128] GBIF.org (2020). GBIF Occurrence Download.

[B5782137] GBIF.org (2020). GBIF Occurrence Download: Palpadaagrorum.

[B5782146] GBIF.org (2020). GBIF Occurrence Download: Microdonaurulentus.

[B5829795] GBIF.org (2020). GBIF Occurrence Download.

[B5983149] Gotelli Nicholas J., Colwell Robert K. (2001). Quantifying biodiversity: procedures and pitfalls in the measurement and comparison of species richness. Ecology Letters.

[B5781114] Herrera Carlos M. (1987). Components of pollinator "quality": Comparative analysis of a diverse insect assemblage. Oikos.

[B5781989] Herrera Carlos M. (1989). Pollinator abundance, morphology, and flower visitation rate: analysis of the “quantity” component in a plant-pollinator system. Oecologia.

[B5781925] Holloway Beverley A. (1976). Pollen‐feeding in hover‐flies (Diptera: Syrphidae). New Zealand Journal of Zoology.

[B5830691] Howlett B. G., Walker M. K., Rader R., Butler R. C., Newstrom-Lloyd L. E., Teulon D. A.J. (2011). Can insect body pollen counts be used to estimate pollen deposition on pak choi stigmas. New Zealand Plant Protection.

[B5779742] Inouye David W, Larson Brendon M. H., Ssymank Axel, Kevan Peter G. (2015). Flies and flowers III: Ecology of foraging and pollination. Journal of Pollination Ecology.

[B5781134] King Caroline, Ballantyne Gavin, Willmer Pat G. (2013). Why flower visitation is a poor proxy for pollination: measuring single-visit pollen deposition, with implications for pollination networks and conservation. Methods in Ecology and Evolution.

[B5781706] Klecka Jan, Hadrava Jiří, Biella Paolo, Akter Asma (2018). Flower visitation by hoverflies (Diptera: Syrphidae) in a temperate plant-pollinator network. PeerJ.

[B5827629] Larson Jonathan L., Kesheimer Adam J., Potter Daniel A. (2014). Pollinator assemblages on dandelions and white clover in urban and suburban lawns. Journal of Insect Conservation.

[B5781124] Levesque Christine M., Burger John F. (1982). Insects (Diptera, Hymenoptera) associated with *Minuartiagroenlandica* (Caryophyllaceae) on Mount Washington, New Hampshire, U.S.A., and their possible role as pollinators. Arctic and Alpine Research.

[B5781935] Lucas Andrew, Bodger Owen, Brosi Berry J., Ford Col R., Forman Dan W., Greig Carolyn, Hegarty Matthew, Jones Laura, Neyland Penelope J., de Vere Natasha (2018). Floral resource partitioning by individuals within generalised hoverfly pollination networks revealed by DNA metabarcoding. Scientific Reports.

[B5827588] Lunau Klaus (2014). Visual ecology of flies with particular reference to colour vision and colour preferences. Journal of Comparative Physiology A.

[B5827555] Lysenkov S. N. (2009). On the estimation of the influence of the character of insect pollinators movements on the pollen transfer dynamics. Entomological Review.

[B5778737] Miranda G. F.G., Young A. D., Locke M. M., Marshall S. A., Skevington J. H., Thompson F. C. (2013). Key to the genera of Nearctic Syrphidae. Canadian Journal of Arthropod Identification.

[B5574722] Mohlenbrock Robert H. (2014). Vascular Floral of Illinois.

[B5647247] Oksanen Jari, Blanchet F. Guillaume, Friendly Michael, Kindt Roeland, Legendre Pierre, McGlinn Dan, Minchin Peter R., O'Hara R. B., Simpson Gavin L., Solymos Peter, Stevens M. Henry H., Szoecs Eduard, Wagner Helene (2019). vegan: Community Ecology Package. https://CRAN.R-project.org/package=vegan.

[B5827598] Ollerton Jeff, Alarcón Ruben, Waser Nickolas M., Price Mary V., Watts Stella, Cranmer Louise, Hingston Andrew, Peter Craig I., Rotenberry John (2009). A global test of the pollination syndrome hypothesis. Annals of Botany.

[B5804884] Orford Katherine A., Vaughan Ian P., Memmott Jane (2015). The forgotten flies: the importance of non-syrphid Diptera as pollinators. Proceedings of the Royal Society B: Biological Sciences.

[B5781074] Ornduff Robert (1975). Complementary roles of halictids and syrphids in the pollination of *Jepsoniaheterandra* (Saxifragaceae). Evolution.

[B5827565] Rader Romina, Edwards Will, Westcott David A., Cunningham Saul A., Howlett Bradley G. (2011). Pollen transport differs among bees and flies in a human-modified landscape. Diversity and Distributions.

[B6105546] Rader Romina, Bartomeus Ignasi, Garibaldi Lucas A, Garratt Michael P D, Howlett Brad G, Winfree Rachael, Cunningham Saul A, Mayfield Margaret M, Arthur Anthony D, Andersson Georg K S, Bommarco Riccardo, Brittain Claire, Carvalheiro Luísa G, Chacoff Natacha P, Entling Martin H, Foully Benjamin, Freitas Breno M, Gemmill-Herren Barbara, Ghazoul Jaboury, Griffin Sean R, Gross Caroline L, Herbertsson Lina, Herzog Felix, Hipólito Juliana, Jaggar Sue, Jauker Frank, Klein Alexandra-Maria, Kleijn David, Krishnan Smitha, Lemos Camila Q, Lindström Sandra A M, Mandelik Yael, Monteiro Victor M, Nelson Warrick, Nilsson Lovisa, Pattemore David E, Pereira Natália de O, Pisanty Gideon, Potts Simon G, Reemer Menno, Rundlöf Maj, Sheffield Cory S, Scheper Jeroen, Schüepp Christof, Smith Henrik G, Stanley Dara A, Stout Jane C, Szentgyörgyi Hajnalka, Taki Hisatomo, Vergara Carlos H, Viana Blandina F, Woyciechowski Michal (2015). Non-bee insects are important contributors to global crop pollination.. Proceedings of the National Academy of Sciences of the United States of America.

[B5793627] Raguso Robert A. (2020). Don’t forget the flies: dipteran diversity and its consequences for floral ecology and evolution. Applied Entomology and Zoology.

[B5829603] Reemer Menno, Rotheray Graham E. (2009). Pollen feeding larvae in the presumed predatory syrphine genus *Toxomerus* Macquart (Diptera, Syrphidae). Journal of Natural History.

[B5781970] Rotheray Graham E., Gilbert Francis S. (2011). The natural history of hoverflies.

[B5781716] Scribailo Robin W., Posluszny Usher (1984). The reproductive biology of Hydrocharis morsus-ranae. I. Floral biology. Canadian Journal of Botany.

[B5556934] Skevington Jeffrey H., Locke Michelle M., Young Andrew D., Moran Kevin, Crins William J., Marshall Stephen A. (2019). Field guide to the flower flies of Northeastern North America.

[B5781901] Ssymank A., Gilbert F. (1993). Anemophilous pollen in the diet of Syrphid flies with special reference to the leaf feeding strategy occurring in Xylotini. (Diptera, Syrphidae). Deutsche Entomologische Zeitschrift.

[B5781776] Ssymank Axel, Kearns C. A., Pape Thomas, Thompson F. Christian (2008). Pollinating flies (Diptera): A major contribution to plant diversity and agricultural production. Biodiversity.

[B5782009] Stein Bruce A., Kutner Lynn S., Adams Jonathan S. (2000). Precious heritage: The status of biodiversity in the United States.

[B5781094] Tepedino V. J., Bowlin W. R., Griswold T. L. (2011). Diversity and pollination value of insects visiting the flowers of a rare buckwheat (*Eriogonumpelinophilum*: Polygonaceae) in disturbed and "natural" areas. Journal of Pollination Ecology.

[B5818621] Udy Kristy L., Reininghaus Hannah, Scherber Christoph, Tscharntke Teja (2020). Plant–pollinator interactions along an urbanization gradient from cities and villages to farmland landscapes. Ecosphere.

[B5782074] Woods A. J., Omernik J. M., Pederson C. L., Moran B. C. (2006). Level III and IV ecoregions of Illinois. EPA/600/R-06/104..

